# Comorbidity and Prognosis in Octogenarians with Infective Endocarditis

**DOI:** 10.3390/jcm11133774

**Published:** 2022-06-29

**Authors:** Jose-Angel Perez-Rivera, Carlos Armiñanzas, Patricia Muñoz, Martha Kestler, Blanca Pinilla, Maria-Carmen Fariñas, Ignacio Alvarez-Rodriguez, Guillermo Cuervo, Angeles Rodriguez-Esteban, Aristides de Alarcón, Andrea Gutiérrez-Villanueva, Ana Pello-Lazaro, Manuel Martínez Sellés, on behalf of the Spanish Collaboration on Endocarditis—Grupo de Apoyo al Manejo de la Endocarditis Infecciosa en ESpaña (GAMES)

**Affiliations:** 1 Unidad Especializada de Insuficiencia Cardiaca, Servicio de Cardiología, Hospital Universitario de Burgos, Universidad Isabel I, 09003 Burgos, Spain; jangel.perezrivera@secardiologia.es; 2 Servicio de Enfermedades Infecciosas Hospital Universitario Marqués de Valdecilla IDIVAL, 39008 Santander, Spain; carlos.arminanzas@scsalud.es; 3 Servicio de Microbiología Clínica y Enfermedades Infecciosas, Hospital General Universitario Gregorio Marañón, Instituto de Investigación Sanitaria Gregorio Marañón, CIBER Enfermedades Respiratorias-CIBERES (CB06/06/0058), Facultad de Medicina, Universidad Complutense de Madrid, 28040 Madrid, Spain; pmunoz@micro.hggm.es; 4 Servicio de Microbiología Clínica y Enfermedades Infecciosas, Hospital General Universitario Gregorio Marañón, Instituto de Investigación Sanitaria Gregorio Marañón, 28007 Madrid, Spain; kestler.martha@gmail.com; 5 Servicio de Medicina Interna, Hospital General Universitario Gregorio Marañón, 28007 Madrid, Spain; blancapinilla@telefonica.net; 6 Servicio de Enfermedades Infecciosas Hospital Universitario Marqués de Valdecilla IDIVAL, CIBER de Enfermedades Infecciosas-CIBERINFEC (CB21/13/00068), Instituto de Salud Carlos III, Universidad de Cantabria, 39008 Santander, Spain; mcarmen.farinas@scsalud.es; 7 Servicio de Enfermedades Infecciosas, Hospital Donostia, OSI Donostialdea, 20014 San Sebastián, Spain; ignacioalvarezrod@gmail.com; 8 Servicio de Enfermedades Infecciosas, Hospital Clinic-IDIBAPS, Universidad de Barcelona, 08036 Barcelona, Spain; glcuervo@clinic.cat; 9 Servicio de UCI Cirugía Cardiaca, Hospital Central de Asturias, 33011 Oviedo, Spain; angelesresteban@gmail.com; 10 Clinical Unit of Infectious Diseases, Microbiology, and Preventive Medicine Infectious Diseases Research Group Institute of Biomedicine of Seville (IBiS), University of Seville/CSIC/University Hospital Virgen del Rocío, 41013 Seville, Spain; aa2406ge@yahoo.es; 11 Unidad de Enfermedades Infecciosas, Servicio de Medicina Interna, Universitario Puerta de Hierro, Majadahonda, 28222 Madrid, Spain; a.gutierrezv@hotmail.com; 12 Servicio de Cardiología, Unidad de Hospitalización, IIS-Fundación Jiménez Díaz, 28040 Madrid, Spain; ampello@quironsalud.es; 13 Servicio de Cardiología, Hospital General Universitario Gregorio Marañón, CIBERCV, Universidad Complutense, 28040 Madrid, Spain; 14 Facultad de Medicina, Universidad Europea, 28670 Madrid, Spain

**Keywords:** endocarditis, age, Charlson index, comorbidity, surgery

## Abstract

Background. Infective endocarditis (IE) in older patients is associated with a high morbidity, mortality, and functional impairment. The purpose of this study was to describe the current profile of IE in octogenarians and to analyze the prognostic impact of baseline comorbidities in this population. Methods. Patients ≥ 80 years and definite IE from the Spanish IE Prospective Database were included. The effect of Charlson Comorbidity Index (CCI) on in-hospital and 12-month mortality was analyzed. Results. From 726 patients, 357 (49%) had CCI ≥ 3 and 369 (51%) CCI < 3. A total of 265 patients (36.6%) died during hospital admission and 338 (45.5%) during 1-year follow-up. CCI ≥ 3 was an independent predictor of in-hospital and 1-year mortality (odds ratio 1.46, 95% confidence interval 1.07–1.99, *p* = 0.017; hazard ratio 1.34, 95% confidence interval 1.08–1.66, *p* = 0.007, respectively). Surgical management was less common in patients with high comorbidity (CCI ≥ 3 68 [19.0%] vs. CCI < 3 112 ((30.4%) patients, *p* < 0.01). From 443 patients with surgical indication, surgery was only performed in 176 (39.7%). Patients with surgical indication treated conservatively had higher mortality than those treated with surgery (in-hospital mortality: 147 (55.1%) vs. 55 (31.3%), *p* < 0.001), (1-year mortality: 172 (64.4%) vs. 68 [38.6%], *p* < 0.001). Conclusion. About half of octogenarians with IE had high comorbidity with CCI ≥ 3. CCI ≥ 3 was a strong independent predictor of in-hospital and 1-year mortality. Our data suggest that the underperformance of cardiac surgery in this group of patients might have a role in their poor prognosis.

## 1. Introduction

Infective endocarditis (IE) is a rare disease, but its incidence seems to be increasing, especially in older adults [1]. This seems to be related to more frequent healthcare contacts and a higher prevalence of cardiovascular implantable devices and prosthetic heart valves [2]. Healthcare-associated and nosocomial IE are frequently due to multi-resistant pathogens [3]. This fact, and the high prevalence of comorbidities in the elderly [4], explains why IE in advanced age has a poor prognosis [5] and frequent functional impairment [6]. This is particularly true for frail elderly in patients with previous comorbidities [7].

IE management frequently differs according to age group, with a low rate of cardiac surgery in octogenarians [8]. This fact is explained, in part, to the increase in surgical risk seen in advanced ages [9]. In patients undergoing surgery for IE, mortality increases significantly with age and perioperative mortality is about 20% in patients > 75 years [10]. In some cases, as in mitral valve surgery and multiple valve interventions, surgical mortality is even higher [10].

However, the underuse of cardiac surgery is associated with adverse outcomes in older patients with IE [11]. It is unclear if this is mainly due to patient selection and to the influence of comorbidities and functional status in the prognosis but recent data suggest that surgery is underused in elderly patients [12]. As elderly patients are increasingly afflicted with IE, the decision whether to perform surgery or not is very relevant. Surgery should not be denied on the basis of age alone. Shared decision making and experienced multidisciplinary teams’ evaluation are essential in these complex patients, and we need new data regarding the benefit of IE surgery in the elderly.

The purpose of our study is to describe the current profile of IE in octogenarians and to analyze the prognostic impact of baseline comorbidities in this population.

## 2. Materials and Methods

### 2.1. Study Population

All consecutive patients with definite or possible IE, according to the modified Duke criteria [13], were prospectively included in the Spanish Collaboration on Endocarditis—Grupo de Apoyo al Manejo de la Endocarditis infecciosa en España (GAMES) registry maintained by 39 Spanish hospitals from 2008 to 2020. Multidisciplinary teams, including infectious disease physicians, cardiologists, heart surgeons, echocardiographers, microbiologists, and imaging specialists, completed standardized case report forms with IE episode and follow-up data that included clinical, microbiological, and echocardiographic sections [14,15]. A complete list of GAMES investigators is provided in the Supplementary Materials.

### 2.2. Ethics

The study was performed in accordance with the Declaration of Helsinki. The protocol was reviewed and approved by the Institutional Ethics Committee at all participating hospitals, according to local standards. Informed consent was obtained from each patient.

### 2.3. Definitions and Data Collection

All patients with definite IE [13] aged ≥80 years were included. IE management, including the decision to perform surgery and type of surgery, was done by the local medical team following the 2015 European Society of Cardiology (ESC) recommendations [16]. We used the Charlson comorbidity index (CCI) and divided the sample in two groups with CCI ≥ 3 and CCI < 3 points [17]. Indication of surgical treatment was considered based on the criteria of American Heart Association [18] and ESC guidelines [16]. We also evaluated indications for surgery treatment, whether there was a consultation with the cardiac surgery team and its recommendation, and what were the reasons for not performing surgery.

### 2.4. Statistical Analyses

Quantitative variables were expressed as mean and standard deviation (SD) or medians, and interquartile ranges when a normal distribution was not observed as per the Kolmogorov-Smirnov goodness-of-fit test. Qualitative variables were expressed as frequency and percentage. Statistical analysis was performed using a two-tailed χ^2^ test and a Fisher’s exact test, or an analysis of variance test, as appropriate in each case. In-hospital and 1-year mortality were analyzed using logistic regression and Kaplan-Meyer survival analysis. A multivariable regression model was adjusted to estimate survival rate over time as a function of several covariates (age, sex, CCI ≥ 3 points, mitral IE, implantable device IE, multi-valvular IE). A *p*-value < 0.05 was considered statistically significant. We used SPSS package v19.0 (SPSS Inc., Chicago, IL, USA) and Stata statistical software (Release 11.0, Stata Corporation, College Station, TX, USA).

## 3. Results

A total of 726 patients ≥ 80 years with definite endocarditis were included, 357 (49%) with CCI ≥ 3 and 369 (51%) with CCI < 3 (Table 1). Compared with the group of low comorbidity, octogenarians with CCI ≥ 3 were more frequently male, had a higher rate of *Enterococcus* spp. and lower rate of gram-negative bacilli, and had a higher prevalence of nosocomial/health-care related IE. Surgery indication, surgery, and hospital survival were less common in patients with high comorbidity.

A total of 443 patients had surgical indication, but surgery was only performed in 176 (39.7%) patients, mainly due to a high risk profile with high estimated surgical risk (Table 2). The impact on the surgical performance of baseline conditions of the patients with indication for cardiac surgery is shown in Table 3. Compared with patients treated with surgery, those with surgical indication and conservative management were older (83.7 ± 3.4 vs. 82.2 ± 2.3 years, *p* < 0.001), had more common mitral valve location (137 (51.3%) vs. 52 (29.5%), *p* < 0.001) and presented more frequently CCI ≥ 3 points (140 (52.4%) vs. 66 (37.5%), *p* = 0.002). Patients with surgical indication treated conservatively had higher mortality than those treated with surgery (in-hospital mortality: 147 (55.1%) vs. 55 (31.3%), *p* < 0.001), (1-year mortality: 172 (64.4%) vs. 68 (38.6%), *p* < 0.001).

In the whole sample, a total of 265 patients (36.6%) died during hospital admission. The effect of baseline conditions in in-hospital mortality is shown in Table 4. CCI was an independent predictor of in-hospital mortality (Table 5).

**Figure 1:**
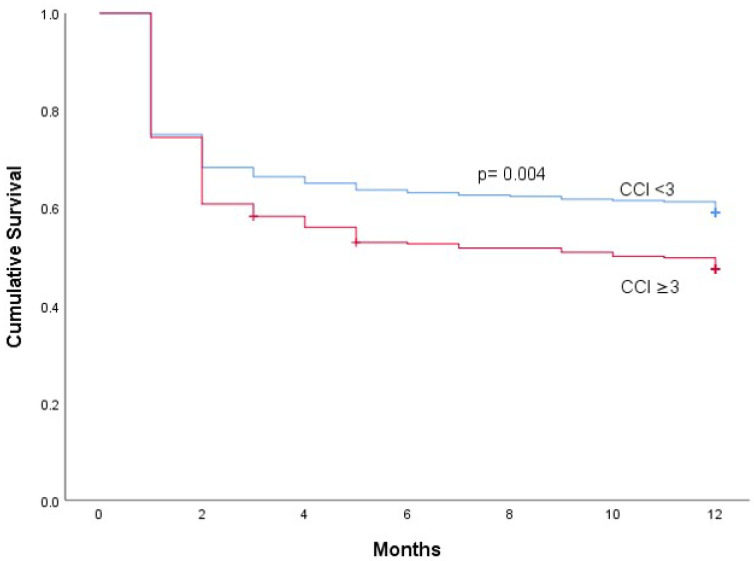
Kaplan-Meier survival according to Charlson Comorbidity Index (CCI).

During 1-year follow-up, 338 (45.5%) patients died. CCI was an independent predictor of in-hospital mortality (Table 6 and Figure 1).

## 4. Discussion

Our main finding is that half of octogenarians with IE had high comorbidity and that a CCI ≥ 3 was a strong predictor of mortality. Our data also suggest that the underperformance of cardiac surgery in this group of patients might have a role in their poor prognosis.

Octogenarians represent a heterogeneous group but usually present an elevated prevalence of predisposing IE events such as previous interventions and recurring health care contacts [2,3,4,5,6,7,8,9,10,11,12,13,14,15,16,17,18,19]. These factors increase the risk of bacteremia with resistant microorganisms [20]. This is particularly true for older patients with comorbidities, as our data show.

The most frequent microorganisms in our sample were *Enterococcus* spp., especially in octogenarians with high comorbidity, and *Streptococcus* spp. in accordance with previous findings [21]. Concomitant diseases like cancer, and associated interventions might explain some of the etiology differences according to the presence of comorbidity. *Enterococcus* spp., especially *Enterococcus faecalis*, is an important cause of IE. This etiology is frequently related to digestive tract conditions, cardiac devices implantation, and vascular access. All these risk factors are more common in the elderly, for instance, the average age at the time of diagnosis for colon cancer is 70 years. The prevalence of enterococcal IE is increasing in recent decades and this is mainly due to population ageing, the increasing number of health care-associated interventions, and microbiological resistance [22]. In previous studies, *Enterococcus* spp. is already the main cause of IE in the elderly [23]

IE patients with advanced age that present are less likely to be operated than younger patients, despite the described lower mortality in patients treated with surgery [11,24,25]. Although older adults have a high risk of in-hospital mortality after surgery, after discharge their mid-term outcomes are similar to the ones seen in younger populations [11,26]. Surgery is associated with lower incidence of adverse events irrespective of age, but it is usually underused in older patients [12].

ESC guidelines recommend assessing comorbidities and operative risk to guide the decision in patients with surgical indication [16]. Comorbidities were strongly connected with in-hospital mortality and with conservative management, in concordance with previous studies [1,5,6,27]. Surgical risk assessment might be done with non-specific risk scores like EuroSCORE II [28] or Society of Thoracic Surgeons (STS) [29], although specific scores for patients with IE have been developed, such as Prosthetic valve, Age ≥ 70, Large intra-cardiac destruction, *Staphylococcus* spp, Urgent surgery, Sex [female], EuroSCORE (PALSUSE) [14], RISK-E score [30], AEPEI score [31] or EndoSCORE [32]. In any case, most of these scores do not consider important geriatric factors such as global comorbidity, frailty, malnutrition and functionality [33].

The Elderly IE study [6] described the functional impact of IE in older patients with IE, suggesting that the management of older patients with IE should include more than antibiotics and surgical decisions. The prevention and treatment of recurrent complications such as delirium, malnutrition, functional decline and drug adverse effects should also be considered. EI teams seem to improve early diagnosis and survival [34]. Incorporating geriatricians and geriatric-expertise cardiologists in these teams could facilitate individualized management of octogenarians [35].

In-hospital mortality in our octogenarian cohort was very high (36.5%). A recent analysis of the Swedish Registry of Infective Endocarditis (SRIE) found that patients ≥ 85 years had an in-hospital mortality of 23% [12]. The high prevalence of comorbidity in our patients might have contributed to this difference. Moreover, in-hospital mortality rates above 20% have been reported in younger patients in previous studies, as in patients > 65 years [3] or >75 years [10]. In addition, recent data published in elderly patients with IE have shown even higher in-hospital mortality than that seen in our cohort [23].

Our study has some limitations. Some recordings of clinical or diagnostic characteristics might be influenced by interobserver variability. In addition, although most GAMES centers have a Cardiac Surgery Department, this is not true for all hospitals. Finally, our data form did not include scales of geriatric syndromes such as frailty and malnutrition. However, our cohort of octogenarians is one of the largest reported and all centers used the same clearly established protocol.

## 5. Conclusions

In conclusion, about half of octogenarians with IE had high comorbidity. CCI ≥ 3 was a strong predictor of mortality. Our data suggest that the underperformance of cardiac surgery in this group of patients might have a role in the poor prognosis of octogenarian patients with high comorbidity. Further studies are needed to describe the role of preoperative multidisciplinary evaluation in comorbid octogenarians with IE.

## Figures and Tables

**Table 1:** Baseline characteristics according to Charlson Comorbidity Index (CCI).

Variables (%)	CCI ≥ 3 (357)	CCI < 3 (369)	*p*
Age (years old). Median (IQR)	83 (81–85)	83 (81–85)	0.835
Sex (Men)	228 (63.8)	197 (53.3)	0.004
Localization			
Aortic	182 (51.0)	202 (54.7)	0.310
Mitral	160 (44.8)	164 (44.4)	0.919
Tricuspid	12 (3.4)	13 (3.5)	0.905
Implantable devices	45 (12.6)	37 (10.0)	0.273
Multi-valvular	53 (14.8)	53 (14.4)	0.854
Native valve	208 (58.3)	227 (61.5)	0.371
Prosthetic valve	120 (33.6)	116 (31.4)	0.531
Comorbidities			
Chronic pulmonary disease	113 (31.6)	46 (12.4)	<0.001
Coronary arterial disease	146 (40.9)	76 (20.6)	<0.001
Congestive heart failure	210 (58.8)	107 (28.9)	<0.001
Diabetes mellitus	159 (44.5)	79 (21.4)	<0.001
Cerebrovascular disease	93 (26.0)	40 (10.8)	<0.001
Neoplasm	135 (37.8)	29 (2.8)	<0.001
Renal insufficiency	181 (50.7)	93 (25.2)	<0.001
Etiology			
*S. aureus*	77 (21.6)	85 (23.0)	0.635
*CNS*	52 (14.6)	66 (17.9)	0.225
*Enterococcus* spp.	85 (23.8)	59 (16.0)	0.008
*Streptococcus* spp.	103 (28.9)	109 (29.5)	0.839
Gram-negative bacilli	9 (2.5)	21 (5.7)	0.032
Site of acquisition			
Community	210 (58.8)	252 (68.3)	0.008
Nosocomial	114 (31.9)	99 (26.8)	0.131
Health care related	33 (9.2)	18 (4.9)	0.021
Clinical course			
Surgical indication	206 (57.7)	237 (64.2)	0.026
Surgery performed	68 (19.0)	112 (30.4)	<0.01
Days of admission. Median (IQR)	33 (20–50)	32 (17–48)	0.198
Days under antibiotic treatment. Median (IQR)	34 (21–42)	37 (23–45)	0.265
In-hospital death	144 (40.3)	121 (32.8)	0.035

CCI: Charlson Comorbidity Index. IQR: interquartile ranges. CNS: coagulase-negative Staphylococci.

**Table 2:** Main reason for conservative treatment according to Charlson Comorbidity Index among patients with surgical indication but surgery finally not performed.

Reasons for NO Surgery (%)	CCI ≥ 3 (140)	CCI < 3 (127)	*p*
Estimated poor surgical prognosis	88 (62.8)	53 (41.7)	0.001
Estimated poor general prognosis	50 (35.7)	45 (35.4)	0.962
Negative of the surgeon	46 (32.8)	35 (27.5)	0.347
Negative of the patient	35 (25.0)	32 (25.2)	0.970
Complexity of surgery	23 (16.4)	25 (19.6)	0.489
Hemodynamic instability	20 (14.2)	18 (14.1)	0.979
Others	19 (13.5)	26 (20.4)	0.132
Stroke	12 (8.5)	19 (14.9)	0.104
Death prior to surgery	11 (7.8)	8 (6.3)	0.621
Bleeding	6 (4.2)	6 (4.7)	0.863
Emergent surgery no available	1 (0.7)	2 (1.5)	0.505

CCI: Charlson Comorbidity Index.

**Table 3:** Patients with surgical indication. Univariate associations of baseline characteristics with surgery.

	No Surgery (267)	Surgery (176)	*p*
Age (years old). Median (IQR)	83 (81–86)	82 (80–84)	<0.001
Male sex	143 (53.5%)	119 (67.6%)	0.003
COPD	63 (23.6%)	40 (22.7%)	0.832
Coronary artery disease	86 (32.2%)	52 (29.5%)	0.554
Heart failure	126 (47.2%)	70 (39.7%)	0.124
Diabetes	85 (31.8%)	52 (29.5%)	0.610
Previous IE	35 (13.1%)	21 (11.9%)	0.715
Peripheral artery disease	50 (18.7%)	30 (17.0%)	0.653
Stroke	65 (24.3%)	29 (16.4%)	0.048
Cancer	120 (44.9%)	55 (31.3%)	0.004
Chronic kidney disease	63 (23.6%)	40 (22.7%)	0.832
CCI. Median (IQR)	3 (1–4)	2 (1–3)	<0.001
Hospital without cardiac surgery	51 (19.1%)	17 (9.7%)	0.007
Mitral valve location	137 (51.3%)	52 (29.5%)	<0.001

IQR: interquartile range. COPD: Chronic obstructive pulmonary disease. IE: Infective endocarditis. CCI: Charlson comorbidity index.

**Table 4:** Univariate associations of baseline characteristics with in-hospital mortality.

	Alive (461)	Dead (265)	*p*
Age (years old). Median (IQR)	83 (81–85)	82 (81–84)	0.039
Male sex	281 (60.9%)	144 (54.3%)	0.082
COPD	92 (19.9%)	67 (25.2%)	0.095
Coronary artery disease	140 (30.3%)	82 (30.9%)	0.871
Heart failure	188 (40.7%)	129 (48.6%)	0.039
Diabetes	138 (29.9%)	100 (37.7%)	0.031
Previous IE	22 (4.8%)	11 (4.2%)	0.699
Peripheral artery disease	49 (10.6%)	31 (11.7%)	0.658
Stroke	73 (15.8%)	60 (22.6%)	0.022
Cancer	105 (22.7%)	59 (22.2%)	0.874
Chronic kidney disease	146 (31.3%)	128 (48.3%)	<0.001
CCI ≥ 3 points	213 (46.2)	144 (54.3)	0.035
Mitral valve location	188 (40.8)	136 (51.3)	0.006
Implantable device IE	72 (15.6)	24 (9.1)	0.012
Multi-valvular IE	58 (12.6)	48 (18.1)	0.042

IQR: interquartile range. COPD: Chronic obstructive pulmonary disease. IE: Infective endocarditis. CCI: Charlson Comorbidity Index.

**Table 5:** Independent predictors of in-hospital mortality.

Variable	OR (95% CI)	*p*
Male sex	0.74 (0.54–1.00)	0.05
Implantable device IE	0.40 (0.23–0.71)	0.002
Multi-valvular IE	1.60 (1.05–2.45)	0.03
CCI ≥ 3 points	1.46 (1.07–1.99)	0.02

OR: odds ratio. CI: confidence interval. IE: infective endocarditis. CCI: Charlson Comorbidity Index. Multivariable logistic regression adjusted by age, sex, CCI ≥ 3 points, mitral IE, implantable device IE, multi-valvular IE.

**Table 6:** Independent predictors of 1-year mortality.

Variable	HR (95% CI)	*p*
Mitral valve location	1.28 (1.03–1.56)	0.025
Implantable device IE	0.64 (0.43–0.97)	0.034
CCI ≥ 3 points	1.34 (1.08–1.66)	0.007

HR: hazard ratio. CI: confidence interval. IE: infective endocarditis. CCI: Charlson Comorbidity Index. Multivariable Cox regression adjusted by age, sex, CCI ≥ 3 points, mitral IE, implantable device IE.

## Data Availability

Not applicable.

## Supplementary Materials

The following supporting information can be downloaded at: https://www.mdpi.com/article/10.3390/jcm11133774/s1, Members of GAMES.
