# Supplementary material for: Comorbidity and Prognosis in Octogenarians with Infective Endocarditis
Source: J Clin Med. 2022 Jun 29;11(13):3774. doi: 10.3390/jcm11133774 (PMC9267499; doi:10.3390/jcm11133774)
Supplement: Supplementary file 1 [file jcm-11-03774-s001.zip › jcm-1770597-supplementary.pdf]

APPENDIX

**Members of GAMES: Hospital Costa del Sol**, (Marbella): Fernando Fernández Sánchez, José Mª García de Lomas, Gabriel Rosas, Javier de la Torre Lima; **Hospital Universitario de Cruces**, (Bilbao): Elena Bereciartua, María José Blanco Vidal, Roberto Blanco, María Victoria Boado, Marta Campaña Lázaro, Alejandro Crespo, Laura Guio Carrión, Mikel Del Álamo Martínez de Lagos, Gorane Euba Ugarte, Ane Josune Goikoetxea, Marta Ibarrola Hierro, José Ramón Iruretagoyena, Josu Irurzun Zuazabal, Leire López-Soria, Miguel Montejo, Javier Nieto, David Rodrigo, Regino Rodríguez, Yolanda Vitoria, Roberto Voces; **Hospital Universitario Virgen de la Victoria**, (Málaga): Mª Victoria García López, Radka Ivanova Georgieva, Guillermo Ojeda, Isabel Rodríguez Bailón, Josefa Ruiz Morales; **Hospital Universitario Donostia-Poliklínica Gipuzkoa-IIS Biodonostia**, (San Sebastián): Ignacio Álvarez Rodríguez, Harkaitz Azkune Galparsoro, Elisa Berritu Boronat, Mª Jesús Bustinduy Odriozola, Cristina del Bosque Martín, Tomás Echeverría, Alberto Eizaguirre Yarza, Ana Fuentes, Miguel Ángel Goenaga, Muskilda Goyeneche del Río, Ángela Granda Bauza, José Antonio Iribarren, Xabier Kortajarena Urkola, José Ignacio Pérez-Moreiras López, Ainhoa Rengel Jiménez, Karlos Reviejo, Alberto Sáez Berbejillo, Elou Sánchez Haza, Rosa Sebastián Alda, Itziar Solla Ruiz, Irati Unamuno Ugartemendia, Diego Vicente Anza, Iñaki Villanueva Benito, Mar Zabalo Arrieta; **Hospital General Universitario de Alicante**, (Alicante): Rafael Carrasco, Vicente Climent, Patricio Llamas, Esperanza Merino, Joaquín Plazas, Sergio Reus; **Complejo Hospitalario Universitario A Coruña**, (A Coruña): Nemesio Álvarez, José María Bravo-Ferrer, Laura Castelo, José Cuenca, Pedro Llinares, Enrique Miguez Rey, María Rodríguez Mayo, Efrén Sánchez, Dolores Sousa Regueiro; **Complejo Hospitalario Universitario de Huelva**, (Huelva): Francisco Javier Martínez; **Hospital Universitario de Canarias**, (Canarias): Mª del Mar Alonso, Beatriz Castro, Teresa Delgado Melian, Javier Fernández Sarabia, Dácil García Rosado, Julia González González, Juan Lacalzada, Lissete Lorenzo de la Peña, Alina Pérez Ramírez, Pablo Prada Arrondo, Fermín Rodríguez Moreno; **Hospital Regional Universitario de Málaga**, (Málaga): Antonio Plata Ciezar,

José Mª Reguera Iglesias; **Hospital Universitario Central Asturias**, (Oviedo): Víctor Asensi Álvarez, Carlos Costas, Jesús de la Hera, Jonnathan Fernández Suárez, Lisardo Iglesias Fraile, Víctor León Arguero, José López Menéndez, Pilar Mencia Bajo, Carlos Morales, Alfonso Moreno Torrico, Carmen Palomo, Begoña Paya Martínez, Ángeles Rodríguez Esteban, Raquel Rodríguez García, Mauricio Telenti Asensio; **Hospital Clínic-IDIBAPS, Universidad de Barcelona**, (Barcelona): Manuel Almela, Juan Ambrosioni, Manuel Azqueta, Mercè Brunet, Marta Bodro, Ramón Cartañá, Guillermo Cuervo, Carlos Falces, Guillermina Fita, David Fuster, Cristina García de la Mària, Delia García-Pares, Marta Hernández-Meneses, Jaume Llopis Pérez, Francesc Marco, José M. Miró, Asunción Moreno, David Nicolás, Salvador Ninot, Eduardo Quintana, Carlos Paré, Daniel Pereda, Juan M. Pericás, José L. Pomar, José Ramírez, Irene Rovira, Elena Sandoval, Marta Sitges, Dolors Soy, Adrián Téllez, José M. Tolosana, Bárbara Vidal, Jordi Vila; **Hospital General Universitario Gregorio Marañón**, (Madrid): Iván Adán, Juan Carlos Alonso, Ana Álvarez-Uría, Javier Bermejo, Emilio Bouza, Gregorio Cuerpo Caballero, Antonia Delgado Montero, Ramón Fortuny Ribas, Esther Gargallo, Ana González Mansilla, Mª Eugenia García Leoni, Francisco Javier González Moraga,Víctor González Ramallo, Martha Kestler Hernández, Amaia Mari Hualde, Marina Machado, Mercedes Marín, Manuel Martínez-Sellés, Rosa Melero, Patricia Muñoz, Diego Monzón, María Olmedo, Álvaro Pedraz, Blanca Pinilla, Ángel Pinto, Cristina Rincón, Hugo Rodríguez-Abella, Marta Rodríguez-Créixems, Eduardo Sánchez-Pérez, Antonio Segado, Neera Toledo, Maricela Valerio, Pilar Vázquez, Eduardo Verde Moreno, Sofía de la Villa; **Hospital Universitario La Paz**, (Madrid): Isabel Antorrena, Belén Loeches, Mar Moreno, Ulises Ramírez, Verónica Rial Bastón, María Romero, Sandra Rosillo; **Hospital Universitario Marqués de Valdecilla**, (Santander): **Hospital Universitario Marqués de Valdecilla**, (Santander): Jesús Agüero Balbín, Cristina Amado, Carlos Armiñanzas Castillo, Francisco Arnaiz de las Revillas, Manuel Cobo Belaustegui, María Carmen Fariñas, Concepción Fariñas-Álvarez, Marta Fernández Sampedro, Iván García, Claudia González Rico, Laura Gutierrez-Fernandez , Manuel Gutiérrez-Cuadra, José Gutiérrez Díez, Marcos Pajarón, José Antonio Parra, Ramón Teira, Jesús Zarauza; **Hospital Universitario Puerta de Hierro**, (Madrid): Jorge Calderón Parra, Marta Cobo, Fernando Domínguez, Pablo García Pavía,

Ana Fernández Cruz, Antonio Ramos-Martínez, Isabel Sánchez Romero; **Hospital Universitario Ramón y Cajal**, (Madrid): Tomasa Centella, José Manuel Hermida, José Luis Moya, Pilar Martín-Dávila, Enrique Navas, Enrique Oliva, Alejandro del Río, Jorge Rodríguez-Roda Stuart, Soledad Ruiz; **Hospital Universitario Virgen de las Nieves**, (Granada): Carmen Hidalgo Tenorio; **Hospital Universitario Virgen Macarena**, (Sevilla): Manuel Almendro Delia, Omar Araji, José Miguel Barquero, Román Calvo Jambrina, Marina de Cueto, Juan Gálvez Acebal, Irene Méndez, Isabel Morales, Luis Eduardo López-Cortés; **Hospital Universitario Virgen del Rocío**, (Sevilla): Arístides de Alarcón, Encarnación Gutiérrez-Carretero, José Antonio Lepe, José López-Haldón, Rafael Luque-Márquez, Guillermo Marín, Antonio Ortiz-Carrellán, Eladio Sánchez-Domínguez; **Hospital San Pedro**, (Logroño): Luis Javier Alonso, Pedro Azcárate, José Manuel Azcona Gutiérrez, José Ramón Blanco, Antonio Cabrera Villegas, Lara García-Álvarez, Concepción García García, José Antonio Oteo; **Hospital de la Santa Creu i Sant Pau**, (Barcelona): Natividad de Benito, Mercé Gurguí, Cristina Pacho, Roser Pericas, Guillem Pons; **Complejo Hospitalario Universitario de Santiago de Compostela**, (A Coruña): M. Álvarez, A. L. Fernández, Amparo Martínez, A. Prieto, Benito Regueiro, E. Tijeira, Marino Vega; **Hospital Universitario Araba**, (Vitoria): Amaia Aguirre Quiñonero, Ángela Alonso Miñambres, Juan Carlos Gainzarain Arana, Sara González de Alaiza Ortega, Miguel Ángel Morán Rodríguez, Anai Moreno Rodríguez, Zuriñe Ortiz de Zárate, José Joaquín Portu Zapirain, Ester Sáez de Adana Arroniz, Daisy Carolina Sorto Sánchez; **Hospital SAS Línea de la Concepción**, (Cádiz): Sánchez-Porto Antonio, Úbeda Iglesias Alejandro; **Hospital Clínico Universitario Virgen de la Arrixaca** (Murcia): José Mª Arribas Leal, Elisa García Vázquez, Alicia Hernández Torres, Ana Blázquez, Gonzalo de la Morena Valenzuela; **Hospital de Txagorritxu**, (Vitoria): Ángel Alonso, Javier Aramburu, Felicitas Elena Calvo, Anai Moreno Rodríguez, Paola Tarabini-Castellani; **Hospital Virgen de la Salud,** (Toledo): Eva Heredero Gálvez, Carolina Maicas Bellido, José Largo Pau, Mª Antonia Sepúlveda, Pilar Toledano Sierra, Sadaf Zafar Iqbal-Mirza; **Hospital Rafael Méndez,** (Lorca-Murcia):, Eva Cascales Alcolea, Ivan Keituqwa Yañez, Julián Navarro Martínez, Ana Peláez Ballesta; **Hospital Universitario San Cecilio** (Granada): Eduardo Moreno Escobar, Alejandro Peña Monje, Valme Sánchez Cabrera, David

Vinuesa García; **Hospital Son Llátzer** (Palma de Mallorca): María Arrizabalaga Asenjo, Carmen Cifuentes Luna, Juana Núñez Morcillo, Mª Cruz Pérez Seco, Aroa Villoslada Gelabert; **Hospital Universitario Miguel Servet** (Zaragoza): Carmen Aured Guallar, Nuria Fernández Abad, Pilar García Mangas, Marta Matamala Adell, Mª Pilar Palacián Ruiz, Juan Carlos Porres; **Hospital General Universitario Santa Lucía** (Cartagena): Begoña Alcaraz Vidal, Nazaret Cobos Trigueros, María Jesús Del Amor Espín, José Antonio Giner Caro, Roberto Jiménez Sánchez, Amaya Jimeno Almazán, Alejandro Ortín Freire, Monserrat Viqueira González; **Hospital Universitario Son Espases** (Palma de Mallorca): Pere Pericás Ramis, Mª Ángels Ribas Blanco, Enrique Ruiz de Gopegui Bordes, Laura Vidal Bonet; **Complejo Hospitalario Universitario de Albacete** (Albacete): Mª Carmen Bellón Munera, Elena Escribano Garaizabal, Antonia Tercero Martínez, Juan Carlos Segura Luque; **Hospital Universitario Terrassa:** Cristina Badía, Lucía Boix Palop, Mariona Xercavins, Sónia Ibars. **Hospital Universitario Dr. Negrín** (Gran Canaria): Xerach Bosch, Eloy Gómez Nebreda, Ibalia Horcajada Herrera, Irene Menduiña Gallego, Imanol Pulido; **Complejo Hospitalario Universitario Insular Materno Infantil** (Las Palmas de Gran Canaria): Héctor Marrero Santiago, Isabel de Miguel Martínez, Elena Pisos Álamo, Daniel San Román Sánchez; **Hospital Universitario 12 de Octubre** (Madrid): Eva Mª Aguilar Blanco, Mercedes Catalán González, María Angélica Corres Peiretti, Andrea Eixerés Esteve, Laura Domínguez Pérez, Santiago de Cossío Tejido, Francisco Galván Román, José Antonio García Robles, Francisco López Medrano, Mª Jesús López Gude, Mª Ángeles Orellana Miguel, Patrick Pilkington, Yolanda Revilla Ostalaza, Juan Ruiz Morales, Sebastián Ruiz Solís, Ana Sabín Collado, Marcos Sánchez Fernández, Javier Solera Rallo, Jorge Solís Martín. **Hospital Universitari de Bellvitge (L'Hospitalet de Llobregat):** Francesc Escrihuela-Vidal, Jordi Carratalà, Inmaculada Grau, Carmen Ardanuy, Dámaris Berbel, José Carlos Sánchez Salado, Oriol Alegre, Alejandro Ruiz Majoral, Fabrizio Sbraga, Arnau Blasco, Laura Gracia Sánchez, Iván Sánchez-Rodríguez. **Hospital Universitario Fundación Jiménez Díaz** (Madrid): Gonzalo Aldamiz, Beatriz Álvarez, Alfonso Cabello Úbeda, Ricardo Fernández Roblas, Rafael Hernández, Victoria Andrea Hortigüela Martín, Andrea Kallmeyer, Cristina Landaeta Kancev, Miguel Morante Ruiz, Miguel Ángel Navas Lobato, Iris

Martínez Alemany, Ana María Pello, Laura Prieto, Marta Tomás Mallebrera. **Hospital Basurto** (Bilbao): Mireia de la Peña Triguero, Ruth Esther Figueroa Cerón, Lara Ruiz Gómez. **Hospital del Mar** (Barcelona): Mireia Ble, Juan Pablo Horcajada Gallego, Antonio José Ginel, Inmaculada López, Alexandra Mas, Antoni Mestres, Lluís Molina, Ramón Serrat, Núria Ribas, Francisca Sánchez, Ana Silverio, Marina Suárez, Luisa Sorlí, Lluís Recasens, Manuel Taurón.
